# Chamber-specific wall thickness features in human atrial fibrillation

**DOI:** 10.1098/rsfs.2023.0044

**Published:** 2023-12-15

**Authors:** Jichao Zhao, James Kennelly, Aaqel Nalar, Anuradha Kulathilaka, Roshan Sharma, Jieyun Bai, Ning Li, Vadim V. Fedorov

**Affiliations:** ^1^ Auckland Bioengineering Institute, University of Auckland, Auckland, New Zealand; ^2^ Department of Physiology and Cell Biology, Bob and Corrine Frick Center for Heart Failure and Arrhythmia, The Ohio State University Wexner Medical Center, Columbus, OH, USA

**Keywords:** atrial fibrillation, atrial wall thickness, human atria, cardiac arrhythmogenesis

## Abstract

Persistent atrial fibrillation (AF) is not effectively treated due to a lack of adequate tools for identifying patient-specific AF substrates. Recent studies revealed that in 30–50% of patients, persistent AF is maintained by localized drivers not only in the left atrium (LA) but also in the right atrium (RA). The chamber-specific atrial wall thickness (AWT) features underlying AF remain elusive, though the important role of AWT in AF is widely acknowledged. We aimed to provide direct evidence of the existence of distinguished RA and LA AWT features underlying AF drivers by analysing functionally and structurally mapped human hearts *ex vivo*. Coronary-perfused intact human atria (*n* = 7, 47 ± 14 y.o.; two female) were mapped using panoramic near-infrared optical mapping during pacing-induced AF. Then the hearts were imaged at approximately 170 µm^3^ resolution by 9.4 T gadolinium-enhanced MRI. The heart was segmented, and 3D AWT throughout atrial chambers was estimated and analysed. Optical mapping identified six localized RA re-entrant drivers in four hearts and four LA drivers in three hearts. All RA AF drivers were anchored to the pectinate muscle junctions with the crista terminalis or atrial walls. The four LA AF drivers were in the posterior LA. RA (*n* = 4) with AF drivers were thicker with greater AWT variation than RA (*n* = 3) without drivers (5.4 ± 2.6 mm versus 5.0 ± 2.4 mm, *T*-test *p* < 0.05; *F*-test *p* < 0.05). Furthermore, AWT in RA driver regions was thicker and varied more than in RA non-driver regions (5.1 ± 2.5 mm versus 4.4 ± 2.2 mm, *T*-test *p* < 0.05; *F*-test *p* < 0.05). On the other hand, LA (*n* = 3) with drivers was thinner than the LA (*n* = 4) without drivers. In particular, LA driver regions were thinner than the rest of LA regions (3.4 ± 1.0 mm versus 4.2 ± 1.0 mm, *T*-test *p* < 0.05). This study demonstrates chamber-specific AWT features of AF drivers. In RA, driver regions are thicker and have more variable AWT than non-driver regions. By contrast, LA drivers are thinner than non-drivers. Robust evaluation of patient-specific AWT features should be considered for chamber-specific targeted ablation.

## 1. Introduction

Atrial fibrillation (AF) is the most common sustained heart rhythm disturbance, and is associated with substantial morbidity and mortality [1]. Pulmonary vein ablation is effective for paroxysmal AF, but less for persistent or long-standing persistent AF [2]. Clinical studies identify localized extra-pulmonary AF sources (drivers), which can maintain AF and represent effective ablation targets [1,3]. However, identifying localized AF drivers in the complex three-dimensional (3D) human atrial structure is extremely challenging [4]. The known atrial structural substrates include fibrosis, myofibre orientation and atrial wall thickness (AWT). Most existing studies focused on atrial fibrosis and myofibers, while AWT received less attention [5–7].

The right atrium (RA) and left atrium (LA) may have different AF mechanisms due to their unique anatomy and structures [8]. In the human heart, the RA has larger and more prominent muscle bundle structures, i.e. the crista terminalis (CT) and the pectinate muscles (PM), compared with the LA. Many studies have reported arrhythmogenesis in the lateral RA [6,9–11]. Wu *et al*. [10] demonstrated that a critical PM bundle thickness in the RA may explain why the onset of atrial flutter is often preceded by AF. The complex myo-bundle structures of the RA may create a frequency-dependent sink-to-source mismatch effect [9].

Though the role of the AWT in AF, primarily due to varying myo-bundle structure, is widely acknowledged, its chamber-specific features underlying AF drivers remain elusive [6]. In this study, we will use our 3D structural analysis approaches on seven explanted human hearts to systematically analyse the structural features of 3D AWT underlying AF drivers. The used explanted human hearts were optically mapped using multiple high-resolution CMOS cameras and structurally imaged at up to 170 µm^3^ using high-resolution 9.4 T contrast-enhanced (CE)-MRI.

## 2. Methods

### 2.1. Optical and structural mapping of intact human hearts *ex vivo*

Detailed approaches for recovering explanted human hearts and functional and structural imaging of the atria were described previously [6,12]. Intact explanted human donor hearts (*n* = 7, 47 ± 14 y.o., two females) with AF history and/or cardiac co-morbidities were obtained from Lifeline of Ohio Organ Procurement Organization (table 1). The Institutional Review Board defined the study on samples from deceased donors as Not Human Subjects Research. The entire atria were isolated, coronary perfused, immobilized with blebbistatin and stained with the voltage-sensitive dye di-4ANDBQBS for near-infrared optical mapping (UltimaL CMOS camera, SciMedia, Japan) [4,6]. Induction of AF by burst pacing (2–10 s) from the superior RA and/or posterior LA was attempted in all hearts. AF drivers were defined as localized sites with the most repetitive and recurrent re-entrant pattern that led to neighbouring regions and had a minimum temporal stability of 30%. After the functional mapping, CE-MRI was performed using a 9.4 T Bruker BioSpin spectrometer (Ettlingen, Germany) [4,6].

### 2.2. Structural analysis of human atria

We used a robust computational pipeline to automatically calculate the 3D AWT [8,12]. The atrial geometry was segmented from the 3D CE-MRIs, and then interpolated and smoothed. The epicardial and endocardial surfaces were then separated using a multi-planar convex hull approach by obtaining the smallest convex volume that contained the atrial tissue [12]. The atrial surfaces were treated as boundary conditions where a Laplace equation was solved to delineate LA and RA. A coupled partial differential equation approach by coupling the Laplace equation with two surface trajectory functions was used to estimate the 3D AWT separately for LA and RA [8]. This approach for 3D AWT was more effective than the Laplace approach [13–15]. AWT variance map was constructed at each voxel by calculating the local variance of AWT within a three-pixel radius.

### 2.3. Statistical analysis

Data are presented as mean ± standard deviation, median and the 25th and 75th percentiles’ values of the distribution. Analysis was conducted using a two-tailed *t*-test for the difference in the mean of AWT and *F*-test for variation of AWT distributions from the hearts. A result is considered statistically significant when the *p*-value is less than 0.05.

## 3. Results

**Figure 1.**
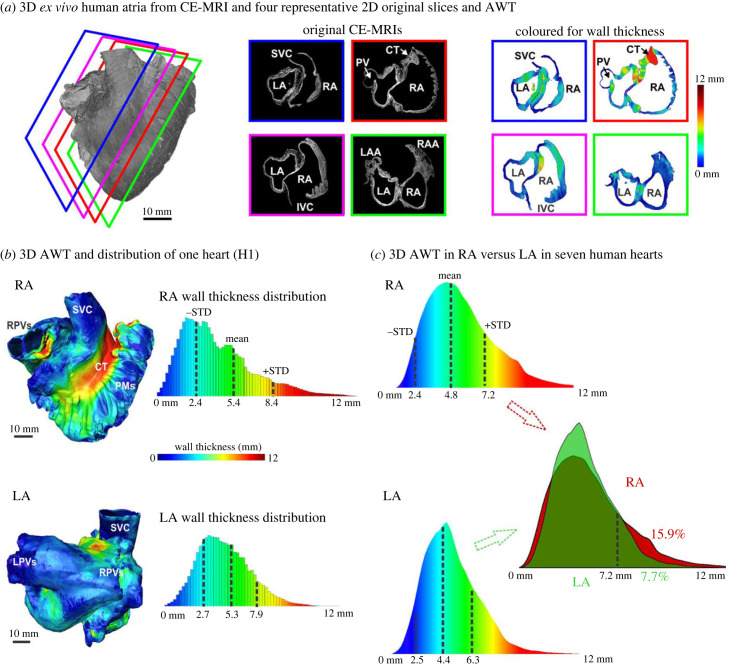
Seven human atria *ex vivo* were imaged by 9.4 T CE-MRI and used for 3D AWT analysis of RA versus LA. (*a*) The human atria (H1) were visualized in 3D at a lateral RA view. Four typical 2D raw CE-MRI images and AWT from the posterior to the anterior of the 3D atria are displayed. Of note, the CT is much thicker than the rest of the tissue. (*b*) Top: The right lateral view of a 3D human RA endocardial surface (heart H1) with colour-coded for AWT. Blue indicates the thin region, while red is for thick atrial tissue. RA AWT distribution is plotted in the right-hand panel. The mean and standard deviation (STD) of the AWT distribution are shown here (black dashed lines). Bottom: The posterior view of 3D human LA endocardial surface (H1) and its AWT distribution. (*c*) The distribution of 3D AWT: RA (top) versus LA (bottom) in the seven explanted human atria. Black dashed lines indicate the mean and STD of the wall thickness distribution. The RA had a secondary peak due to the presence of the CT. In addition, RA had a higher tissue percentage with AWT > 7.2 mm than LA (15.9% versus 7.7%). CE-MRI, contrast-enhanced magnetic resonance imaging; CT, crista terminalis; IAS, interatrial septum; S/IVC, superior/inferior vena cava; LA/RA, left/right atrium; LAA/RAA, left/right atrial appendage; PV, pulmonary vein; L/R PV, left/right superior and inferior PVs; AWT, atrial wall thickness; PM, pectinate muscle; AF, atrial fibrillation.

Segmented human atria were reconstructed in 3D, and four representative two-dimensional (2D) CE-MRIs and estimated AWT from one heart are shown in figure 1*a*. The 3D AWT for the seven human atria *ex vivo* (table 2) showed that the atrial wall was thicker and varied more (RA: 4.8 ± 2.4 mm versus LA: 4.4 ± 1.9 mm, *T*-test *p* < 0.05; *F*-test *p* < 0.05) in the RA than the LA (figure 1*b*,*c*). The RA had a secondary peak due to the presence of the CT. RA had a higher total tissue percentage with AWT > 7.2 mm than LA (15.9% versus 7.7%).

**Table 2.** The statistics of 3D human AWT for RA and LA. It includes the mean, STD, median and the 25th percentiles (Q_25_) and the 75th percentiles (Q_75_) of AWT distribution, as well as atrial chamber cavity and tissue volume. Red and green ticks indicate the RA and LA with optically mapped AF drivers, respectively. RA, right atrium; LA, left atrium; AWT, atrial wall thickness; STD, standard deviation.

heart	H1		H2		H3		H4		H5		H6		H7	
atrium	LA	RA	LA	RA	LA	RA	LA	RA	LA	RA	LA	RA	LA	RA
drivers		✓		✓		✓	✓	✓	✓		✓			
Q_25_ (mm)	3.48	3.41	3.40	3.38	2.84	2.77	2.90	3.51	2.66	2.29	3.24	3.14	2.87	2.68
median (mm)	4.40	4.69	4.50	4.53	3.87	4.27	3.92	5.16	3.62	3.47	4.38	4.43	3.83	3.96
mean (mm)	5.30	5.44	4.51	4.53	3.91	4.48	4.33	5.53	3.62	3.47	4.70	4.76	3.83	3.96
Q_75_ (mm)	6.72	6.82	5.80	5.98	3.87	4.27	5.60	7.21	4.73	5.13	5.66	5.96	4.98	5.99
STD (mm)	2.56	2.91	1.65	2.03	1.47	2.10	1.98	2.66	1.50	2.14	2.14	2.27	1.66	2.19
cavity vol. (cm^3^)	34.99	66.38	32.27	42.16	39.60	36.08	39.16	45.07	34.58	28.96	32.27	42.16	47.10	54.21
tissue vol. (cm^3^)	33.73	47.75	23.66	31.51	24.99	24.11	27.73	34.37	25.68	19.77	24.56	32.59	27.56	22.89

**Figure 2.**
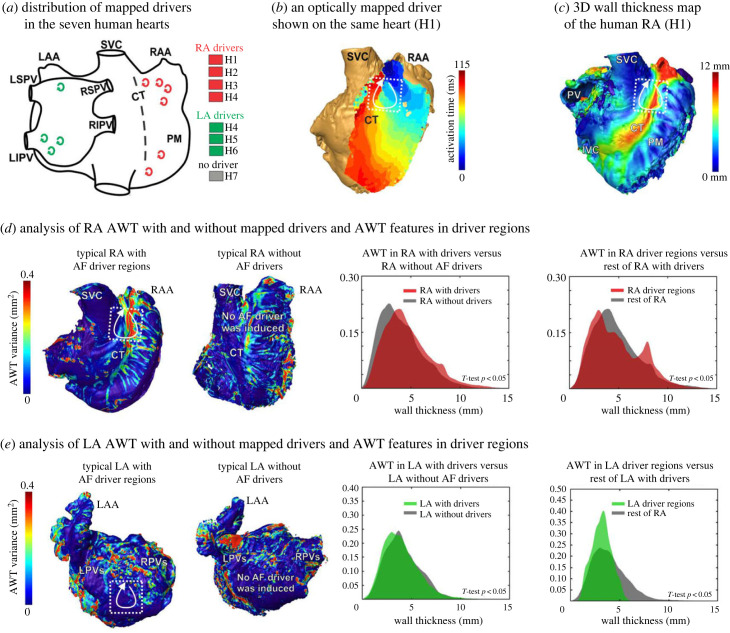
Analysis of chamber-specific AWT with and without mapped AF drivers and AWT features in driver regions in explanted human atria (*n* = 7). (*a*) The distribution of optically mapped AF drivers in LA versus RA for the seven heart hearts. All the RA drivers were anchored on the CT region at both the superior and inferior sections of the RA. (*b*) A typical RA driver of the human atria (H1) is superimposed on the atrial epicardium and co-located with AWT variation (*c*). The white arrow indicates an optically mapped re-entrant driver. It can be seen that this driver is anchored on the thicker (in red) CT and thin (blue) tissue region. (*d*) AWT distribution for RA with and without optically mapped AF drivers, and for RA driver regions and the rest of the RA with AF drivers. RA driver regions specifically had a much larger secondary peak in the distribution due to their co-location with the thick CT regions. (*e*) AWT distribution for LA with and without optically mapped drivers, and for LA driver regions and the rest of the LA. LA driver regions had a much thinner AWT than the rest of the LA tissue. For abbreviations, see figure 1.

Optical mapping revealed that sustained AF (27.0 ± 14.5 min) was induced in six of the seven atrial preparations (table 1). A total of six RA drivers were defined in four hearts (H1–H4) and all were located in the lateral RA close to the CT and PM regions. Four LA drivers were in the posterior LA of the three hearts (H4, H5 and H6), shown in table 1 and figure 2*a*. Particularly, optically mapped RA AF drivers (figure 2*b*) were co-located in the lateral atrial regions with mixed thick (red) and thin (blue) walls (figure 2*c*).

**Table 1.** The characteristics of human hearts *ex vivo* (*N* = 7). RA, right atrium; LA, left atrium; HTN, hypertension; HLD, hyperlipidemia; CHF, congestive heart failure; DMII, diabetes mellitus type II; ERSD, end stage renal disease.

heart no.	H1	H2	H3	H4	H5	H6	H7
sex	male	female	male	female	male	male	male
age	54	24	54	63	58	33	45
conditions	HTN, CHF, DMII, ESRD	asthma, tobacco, alcohol	HTN, HLD, DM, alcohol	HTN, hypothyroidism	tobacco, alcohol	drug abuse, tobacco	HTN, tobacco, alcohol
RA drivers	superior RA; inferior RA	superior RA	superior RA; middle RA	superior RA	none	none	none
LA drivers	none	none	none	posterior LA; posterior LA	posterior LA	posterior LA	none

We conducted a subgroup analysis of AWT depending on whether AF drivers were induced in the atrial chamber, as well as AWT features for the mapped AF driver regions compared with the rest of the chamber regions (figure 2*d*,*e*). Our results indicate that RA with AF drivers was thicker and had a more significant AWT variation (RA with drivers: 5.1 ± 2.5 mm versus RA without drivers: 4.4 ± 2.2 mm, *T*-test *p* < 0.05; *F*-test *p* < 0.05), particularly in the driver regions (RA driver regions: 5.4 ± 2.6 mm versus RA non-driver regions: 5.0 ± 2.4 mm, *T*-test *p* < 0.05; *F*-test *p* < 0.05). RA driver regions specifically had a much larger secondary peak in the distribution due to their co-location with the thick CT regions. On the other hand, LA (*n* = 3) with mapped drivers were thinner (4.2 ± 1.9 mm versus 4.5 ± 1.9 mm, *p* < 0.05) than the LA (*n* = 4) without drivers (figure 2*e*). In addition, LA driver regions (*n* = 4) were thinner than the rest of the LA regions (3.4 ± 1.0 mm versus 4.2 ± 1.0 mm, *p* < 0.05).

## 4. Discussion

The unique strength of this study is that we could access intact human donor hearts with disease history, which were mapped with integrated high-resolution 3D functional and structural approaches [6,12]. These unique data allow us to demonstrate chamber-specific AWT characteristics in AF driver regions. We found (i) atrial wall is thicker with more AWT variance in RA versus LA; (ii) RA with AF drivers are thicker and have greater AWT variation than RA without drivers, particularly in the RA driver regions; (iii) LA driver regions are thinner than the rest of the LA regions; and (iv) LA with AF drivers is thinner than LA without drivers. These results can inform clinicians of the chamber-specific AF driver features for personalized targeted ablation.

It is well established that AWT variation influences the electrical activation patterns, playing an important role in triggering and maintaining AF, particularly in RA [6,12]. A computer modelling study [16] demonstrated that AF drivers drifted towards and anchored to the CT/PMs and the surrounding atrial wall in the RA due to a large source-to-sink mismatch. Interestingly, a recent clinical study indicated that AWT was thinner in LA AF drivers than in non-drivers (2.1 ± 0.2 mm versus 2.23 ± 0.3 mm, *p* < 0.05) in moderated gadolinium-enhanced areas [17]. Notably, their clinical MRI had a relatively low spatial resolution of approximately 1.3 mm which could explain the discrepancy in the LA AWT, compared with our high-resolution MRI results (4.2 ± 1.9 mm versus 4.5 ± 1.9 mm). In addition to AWT, our early studies of the human RA *ex vivo* demonstrated that the intramural fibrotic strands and endo-epicardial myofibre misalignment may create microanatomic tracks for stable re-entrant AF drivers [6,12]. Computational analysis of AF driver features including 3D variations in AWT, transmural fibrosis and myofibres twist may aid in designing personalized ablation strategies.

### 4.1. Study limitations

Only seven *ex vivo* hearts were used in this study. Future studies are necessary to apply the 3D computational high-resolution framework in more human hearts to cover the full range of atrial anatomical variability and remodelling variability. In this study, computed AWT in the LA appendage was affected by its complex anatomy, and thus was not included for analysis. As this study focused on AWT variations along with their association with AF drivers, the influence of fibrosis and myofibre orientation were not included. In addition, this structural analysis study aimed to provide direct evidence of distinct RA and LA AWT features in AF driver regions by analysing functionally and structurally mapped human hearts *ex vivo*. However, an extensive computer modelling analysis is warranted to investigate whether the AWT, its variance/gradient [18], atrial surface curvature [19] or complex PM topology [11] are more important.

## 5. Conclusion

AF driver locations correlate with chamber-specific AWT features in *ex vivo* human hearts. AWT and its variance are important features of AF drivers, particularly in the RA. Future large-scale studies will define metrics of 3D AWT features and their specificity/sensitivity to predict AF drivers as ablation targets.

## Data Availability

Our Matlab computer codes for AWT can be openly accessed using the link: https://doi.org/10.5281/zenodo.10127415 [20].

## References

[1] Lim HS et al. 2017 Complexity and distribution of drivers in relation to duration of persistent atrial fibrillation. J. Am. Coll. Cardiol. **69**, 1257-1269. (10.1016/j.jacc.2017.01.014) 28279292

[2] Brooks AG, Stiles MK, Laborderie J, Lau DH, Kuklik P, Shipp NJ, Hsu L-F, Sanders P. 2010 Outcomes of long-standing persistent atrial fibrillation ablation: a systematic review. Heart Rhythm **7**, 835-846. (10.1016/j.hrthm.2010.01.017) 20206320

[3] Miller JM, Kalra V, Das MK, Jain R, Garlie JB, Brewster JA, Dandamudi G. 2017 Clinical benefit of ablating localized sources for human atrial fibrillation: the Indiana University FIRM Registry. J. Am. Coll. Cardiol. **69**, 1247-1256. (10.1016/j.jacc.2016.11.079) 28279291

[4] Hansen BJ et al. 2018 Human atrial fibrillation drivers resolved with integrated functional and structural imaging to benefit clinical mapping. JACC Clin. Electrophysiol. **4**, 1501-1515. (10.1016/j.jacep.2018.08.024) 30573112 PMC6323649

[5] Zhao J, Butters TD, Zhang H, Pullan AJ, LeGrice IJ, Sands GB, Smaill BH. 2012 An image-based model of atrial muscular architecture: effects of structural anisotropy on electrical activation. Circ. Arrhythm. Electrophysiol. **5**, 361-370. (10.1161/CIRCEP.111.967950) 22423141

[6] Hansen BJ et al. 2015 Atrial fibrillation driven by micro-anatomic intramural re-entry revealed by simultaneous sub-epicardial and sub-endocardial optical mapping in explanted human hearts. Eur. Heart J. **36**, 2390-2401. (10.1093/eurheartj/ehv233) 26059724 PMC4568403

[7] Marrouche NF et al. 2022 Effect of MRI-guided fibrosis ablation vs conventional catheter ablation on atrial arrhythmia recurrence in patients with persistent atrial fibrillation: the DECAAF II randomised clinical trial. J. Am. Med. Assoc. **327**, 2296-2305. (10.1001/jama.2022.8831) PMC9214588 35727277

[8] Wang Y, Xiong Z, Nalar A, Hansen BJ, Kharche S, Seemann G, Loewe A, Fedorov VV, Zhao J. 2019 A robust computational framework for estimating 3D bi-atrial chamber wall thickness. Comput. Biol. Med. **114**, 103444. (10.1016/j.compbiomed.2019.103444) 31542646 PMC6817405

[9] Berenfeld O, Zaitsev AV. 2004 The muscular network of the sheep right atrium and frequency-dependent breakdown of wave propagation. Anat. Rec. A Discov. Mol. Cell Evol. Biol. **280**, 1053-1061. (10.1002/ar.a.20106) 15372488

[10] Wu T-J et al. 1998 Role of pectinate muscle bundles in the generation and maintenance of intra-atrial reentry: potential implications for the mechanism of conversion between atrial fibrillation and atrial flutter. Circ. Res. **83**, 448-462. (10.1161/01.RES.83.4.448) 9721702

[11] Potse M, Gharaviri A, Pezzuto S, Auricchio A, Krause R, Verheule S, Schotten U. 2018 Anatomically-induced fibrillation in a 3D model of the human atria. In 2018 Computing in Cardiology Conf. (CinC), Maastricht, The Netherlands, 23–26 September 2018. (10.22489/CinC.2018.366)

[12] Zhao J et al. 2017 Three dimensional integrated functional, structural, and computational mapping to define the structural ‘fingerprints’ of heart-specific atrial fibrillation drivers in human heart ex vivo. J. Am. Heart Assoc. **6**, e005922. (10.1161/JAHA.117.005922) 28862969 PMC5586436

[13] Bishop M, Rajani R, Plank G, Gaddum N, Carr-White G, Wright M, O'Neill M, Niederer S. 2016 Three-dimensional atrial wall thickness maps to inform catheter ablation procedures for atrial fibrillation. Europace **18**, 376-383. (10.1093/europace/euv073) 25842272 PMC5841557

[14] Karim R et al. 2018 Algorithms for left atrial wall segmentation and thickness—evaluation on an open-source CT and MRI image database. Med. Image Anal. **50**, 36-53. (10.1016/j.media.2018.08.004) 30208355 PMC6218662

[15] Varela M et al. 2017 Novel MRI technique enables non-invasive measurement of atrial wall thickness. IEEE Trans. Med. Imaging **36**, 1607-1614. (10.1109/TMI.2017.2671839) 28422654 PMC5549842

[16] Roy A, Varela M, Aslanidi O. 2018 Image-based computational evaluation of the effects of atrial wall thickness and fibrosis on re-entrant drivers for atrial fibrillation. Front. Physiol. **9**, 1352. (10.3389/fphys.2018.01352) 30349483 PMC6187302

[17] Nakamura T et al. 2022 The impact of the atrial wall thickness in less late-gadolinium enhancement areas on atrial fibrillation drivers in persistent atrial fibrillation patients. J. Arrhythm. **38**, 221-231. (10.1002/joa3.12676) 35387140 PMC8977582

[18] Biktasheva I, Dierckx H, Biktashev VN. 2015 Drift of scroll waves in thin layers caused by thickness features: asymptotic theory and numerical simulations. Phys. Rev. Lett. **114**, 068302. (10.1103/PhysRevLett.114.068302) 25723248

[19] Azzolin L, Luongo G, Ventura SR, Saiz J, Dösse O, Loewe A. 2020 Influence of gradient and smoothness of atrial wall thickness on initiation and maintenance of atrial fibrillation. In 2020 Computing in Cardiology, Rimini, Italy, 13–16 September 2020. (10.22489/CinC.2020.261)

[20] Zhao J, Kennelly J, Nalar A, Kulathilaka A, Sharma R, BaiFedorov J, Li N, Fedorov VV. 2023 Chamber-specific wall thickness features in human atrial fibrillation. Zenodo. (10.5281/zenodo.10127415) PMC10722209 38106912

